# Perinatal post-mortem magnetic resonance imaging (MRI) of the central nervous system (CNS): a pictorial review

**DOI:** 10.1186/s13244-021-01051-0

**Published:** 2021-07-22

**Authors:** Carlos Pérez-Serrano, Álvaro Bartolomé, Núria Bargalló, Carmen Sebastià, Alfons Nadal, Olga Gómez, Laura Oleaga

**Affiliations:** 1 Radiology Department, CDIC, Hospital Clínic de Barcelona, C/Villarroel no. 170, 08036 Barcelona, Spain; 2 Pathology Department, CDB, Hospital Clínic de Barcelona, C/Villarroel no. 170, 08036 Barcelona, Spain; 3 Gynecology Department, ICGON, Hospital Clínic de Barcelona, C/Villarroel no. 170, 08036 Barcelona, Spain; 4 University of Barcelona, Barcelona, Spain

**Keywords:** Foetal magnetic resonance, Central nervous system, Post-mortem, Termination of pregnancy, Ultrasound

## Abstract

Central nervous system (CNS) abnormalities cause approximately 32–37.7% of terminations of pregnancy (TOP). Autopsy is currently the gold standard for assessing dead foetuses and stillborn. However, it has limitations and is sometimes subject to parental rejection. Recent studies have described post-mortem foetal magnetic resonance imaging (MRI) as an alternative and even complementary to autopsy for CNS assessment. Radiologists now play a key role in the evaluation of perinatal deaths. Assessment of foetal CNS abnormalities is difficult, and interpretation of foetal studies requires familiarisation with normal and abnormal findings in post-mortem MRI studies as well as the strengths and limitations of the imaging studies. The purpose of this pictorial review is to report our experience in the post-mortem MRI evaluation of the CNS system, including a description of the protocol used, normal CNS findings related to post-mortem status, abnormal CNS findings in our sample, and the correlation of these findings with histopathological results.

## Key points

- While invasive autopsy is currently the gold standard to evaluate foetuses and stillborn post-mortem, it has some important limitations.
- Post-mortem MRI is useful for the assessment of CNS abnormalities.
- It is essential to be aware of some specific “normal” imaging characteristics in post-mortem MRI in order to avoid misdiagnosis.
- There is high overall concordance between post-mortem MRI and autopsy in detecting foetal and stillborn CNS abnormalities and, therefore, MRI may be a valid alternative when autopsy cannot be performed.
- Routine performance of post-mortem MRI combined with autopsy would be useful for assessing the role of the CNS in perinatal death as the two techniques are complementary.

## Background

Perinatal losses due to unexplained intrauterine deaths or foetal abnormalities or maternal disease are devastating in routine obstetric practice. Despite advances in screening and antenatal care, the stillbirth rate in Europe ranges from 0.2 to 0.5% [1]. It is estimated that major structural or genetic birth defects can affect approximately 3% of births, being major contributors to perinatal mortality [2]. In this regard, some series report that central nervous system (CNS) abnormalities cause approximately 32–37.7% of terminations of pregnancy (TOP) [3, 4].

At present, invasive autopsy is the gold standard for evaluating dead foetuses and stillborn. However, conventional invasive autopsy has some limitations. Small foetuses, and time between foetal death and autopsy, especially in feticide before delivery induction causes autolytic phenomena and maceration, particularly in the brain, sometimes making autopsy and correct evaluation of the CNS a challenge. Furthermore, the foetal or stillborn brain is difficult to manage even with adequate fixation or after brief post-mortem delay [5].

Many parents demand to be informed on the cause of the demise and the risk of abnormalities in future pregnancies [6–10]. However parents also frequently request all organs to be replaced before burial, making adequate fixation unfeasible [8, 9], and in some cases autopsy is denied.

Brain evaluation is critical; foetal brain malformations occur in 2–3 per 1000 pregnancies and are the most frequent reasons for TOP [11].

Imaging techniques are helpful in determining the cause of death when autopsy cannot be performed. Conventional radiography has frequently been used to evaluate post-mortem abnormalities in foetuses and neonates. However, the information provided is scarce and is limited to assessing the chest or bones [12].

Post-mortem ultrasound (US), computed tomography (CT) and magnetic resonance imaging (MRI) are possible alternatives for the assessment of perinatal causes of death.

Post-mortem US examination is a useful widely available technique that is easy to perform. However, post-mortem changes and loss of pliability due to rigor mortis can decrease the accuracy of the technique [13, 14].

Post-mortem CT is useful for the assessment of musculoskeletal abnormalities, but lacks good diagnostic accuracy for brain evaluation [14]. Different studies have shown that post-mortem brain MRI study could be an alternative to autopsy in that it is as accurate as autopsy and provides structural information of the brain in foetuses and stillborn neonates [8, 9, 15–17]. Moreover, MRI is significantly better tolerated by parents than conventional autopsy [18, 19]. Post-mortem MRI also has other advantages such as the ability to assess the brain and spinal cord in situ*,* thereby avoiding possible damage to the anatomy that could occur during dissection. The images obtained can be reviewed and fully reassessed at any time and there are no motion artefacts that often occur in prenatal MRI and can degrade the images [17].

Foetal brain development undergoes different stages showing specific imaging characteristics that differ from those of the brain of a newborn. In addition, the death phenomenon and the whole birth process lead to MRI abnormalities that can be misunderstood as pathological. It is crucial to be aware and interpret all these imaging features in order to avoid misdiagnosis.

The aim of this pictorial review is to show normal and abnormal post-mortem foetal MRI findings, adding the most illustrative cases from our experience. We have retrospectively reviewed 53 post-mortem foetal brain MRI scans (gestational ages between 21 and 41 weeks) performed at our institution between 2012 and 2019, described the protocol performed at our centre for CNS evaluation, described normal and abnormal CNS findings related to post-mortem status, and correlated MRI findings in CNS diseases with neuropathological findings in cases where conventional autopsy was performed.

### MRI protocol

Although there is no clear consensus in the literature on how to perform post-mortem MRI, 3 Tesla (3 T) MRI studies have demonstrated to be more accurate than 1.5 T for evaluating foetal abnormalities [20]. Nonetheless, 3 T MRI equipment is not available in all centres.

MRI protocols performed in previous publications are variable, as some use only T1 and T2 sequences [5, 8], which are the basis for determining anatomy and the majority of morphological alterations. The addition of susceptibility weighted imaging (SWI) sequences is useful for detecting bleeding. On the other hand, diffusion-weighted imaging (DWI) can help to detect cytotoxic oedema, although it may be inaccurate in cases of brain maceration due to autolytic phenomena [21]. Table 1 shows the parameters of the post-mortem 3 T MRI protocol performed at our centre including T1, T2, SWI and DWI sequences.

**Table 1:** MRI protocol. 3-T unit Magnetom Trio Tim (Siemens Medical Solutions, Erlangen, Germany) with 32-channel phased array head coil

	3D Sag t1W	3D Sag T2W	3D Ax CISS	Ep2d_diff_800	SWI
TR	1710 ms	3200 ms	6.8 ms	11,000 ms	28 ms
TE	2.65 ms	394 ms	2.95 ms	163 ms	20 ms
FA	8°	120°	50°	90°	15°
FOV	110 mm	110 mm	110 mm	110 mm	110 mm
Matrix	256 × 256	384 × 384	512 × 512	2 mm	0.57 mm
Thickness	0.88 mm	0.5 mm	0.3 mm	192 × 256	192 × 256

### Normal findings according to gestational age

The different stages of normal foetal development show specific imaging characteristics that may differ from those of a full-term newborn brain.

**Fig. 1:**
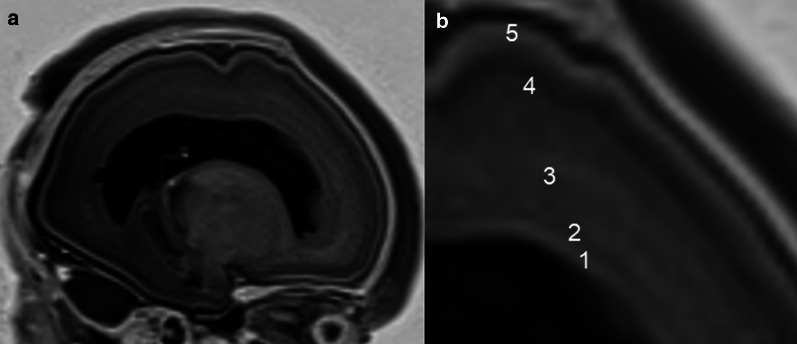
23-week foetus with normal cerebral lamination. **a**, **b** Sagittal T1-weighted images show five laminar compartments: (1) ventricular zone; (2) periventricular zone; (3) intermediate zone; (4) subplate zone; (5) cortical plate

Normal foetal cerebral lamination has been described on T1-weighted (T1W) and T2-weighted images (T2W), from the 19th and 21st gestational weeks, as three laminar compartments: the cortical plate (precursor of the cortex), with high signal intensity on T1W due to high cellularity, the subplate, a band of low signal on T1W, and the ventricular layer, hyperintense on T1W. Between the 21st and 27th gestational weeks, 5 main layers can be identified within the brain parenchyma. Starting from the ventricular surface, these laminar compartments are as follows: (1) The ventricular zone (germinal matrix) hyperintense on T1W images due to its high cellularity; (2) The periventricular zone of low signal intensity on T1W images, which largely corresponds to the periventricular fibre-rich zone (however it may not be clearly visualised in all cerebral regions); (3) The intermediate zone of moderate signal intensity, which encompasses both the subventricular cellular zone and the foetal white matter; (4) The subplate zone of low signal intensity on T1W images; (5) The cortical plate hyperintense on T1W images [22–24] (Fig. 1). Different alterations in foetal brain lamination have been described as potential biomarkers of underlying diseases such as genetic disorders, neuronal migration disorders, and even as a consequence of other acquired disorders such as ventriculomegaly or hypoxic-ischaemic injuries [25]. However, after 26 weeks of gestation, the lamination patterns in the cerebral wall gradually disappear [22].

**Fig. 2.**
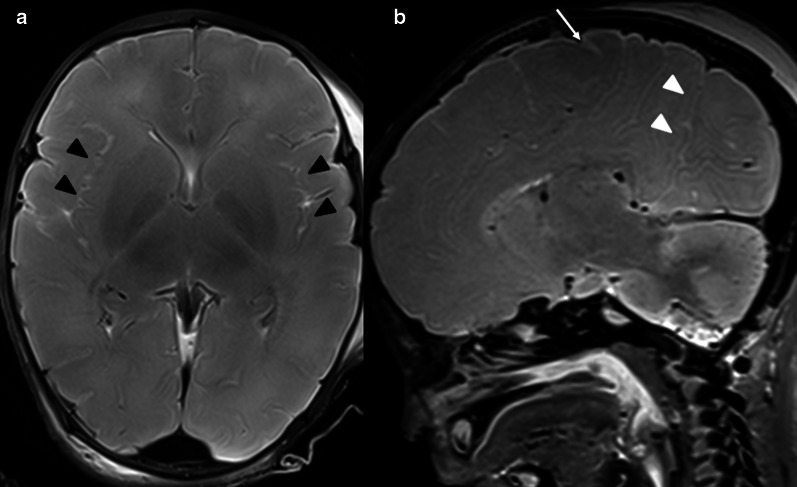
36-week foetus with normal cerebral sulcation. **a** Axial T2-weighted image shows normal Sylvian fissure (black arrowheads). **b** Sagittal T2-weighted image shows normal parieto-occipital fissure (white arrowheads) and central sulcus (white arrow)

The sulcation pattern begins with the development of the Sylvian fissure at approximately 16 weeks, followed by the parieto-occipital fissure at 22 weeks and the central sulcus at 26 weeks and is almost complete by 34 weeks [26, 27] (Fig. 2).

The human cerebellum is one of the first brain structures to differentiate starting in the fourth week of gestation. The greatest growth takes place during the second half of gestation, when a period of active migration and cellular differentiation occur. This results in development of the cerebellar vermis and hemispheres, as well as a more than fivefold increase of cerebellar size between 20 and 40 weeks of gestation [19, 28].

**Fig. 3:**
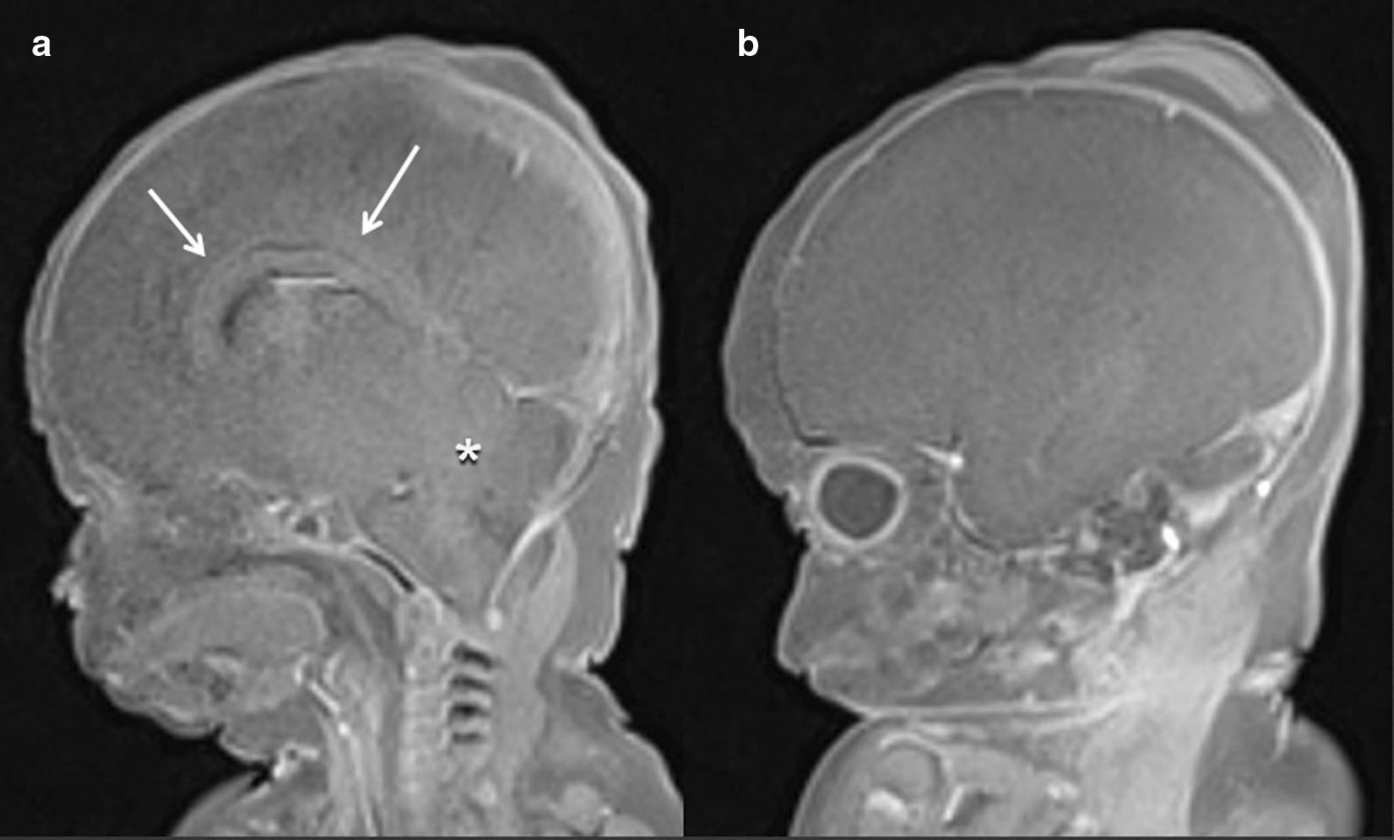
25-week foetus with normal cerebral lamination. **a**, **b** Sagittal T1-weighted images, normal midline corpus callosum (arrows) and posterior fossa (asterisk)

The development of the corpus callosum takes place between the 12th and 20th weeks of gestation. It begins with the anterior body and continues bidirectional, with the anterior portion (genu) developing earlier than the posterior portion (splenium). Myelination of the corpus callosum is produced in the opposite direction, from the splenium forwards [29]. The neonatal corpus callosum is thin in its entirety [27] (Fig. 3).

### Normal post-mortem findings/pshysiological changes

**Fig. 4:**
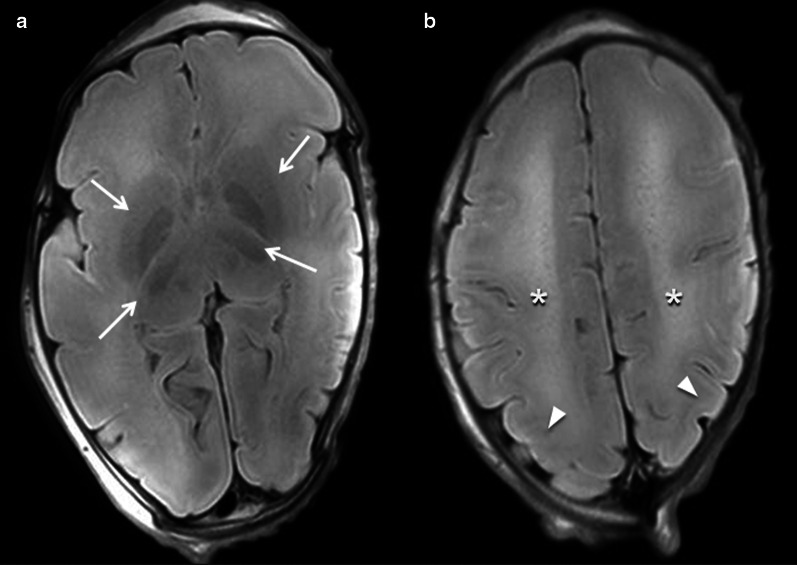
Normal cerebral lamination in a 34-week foetus. Axial T2W images **a** better grey-white matter differentiation, deep grey matter (arrows), **b** cerebral cortex (arrowheads), centrum semiovale (asterisks)

**Fig. 5:**
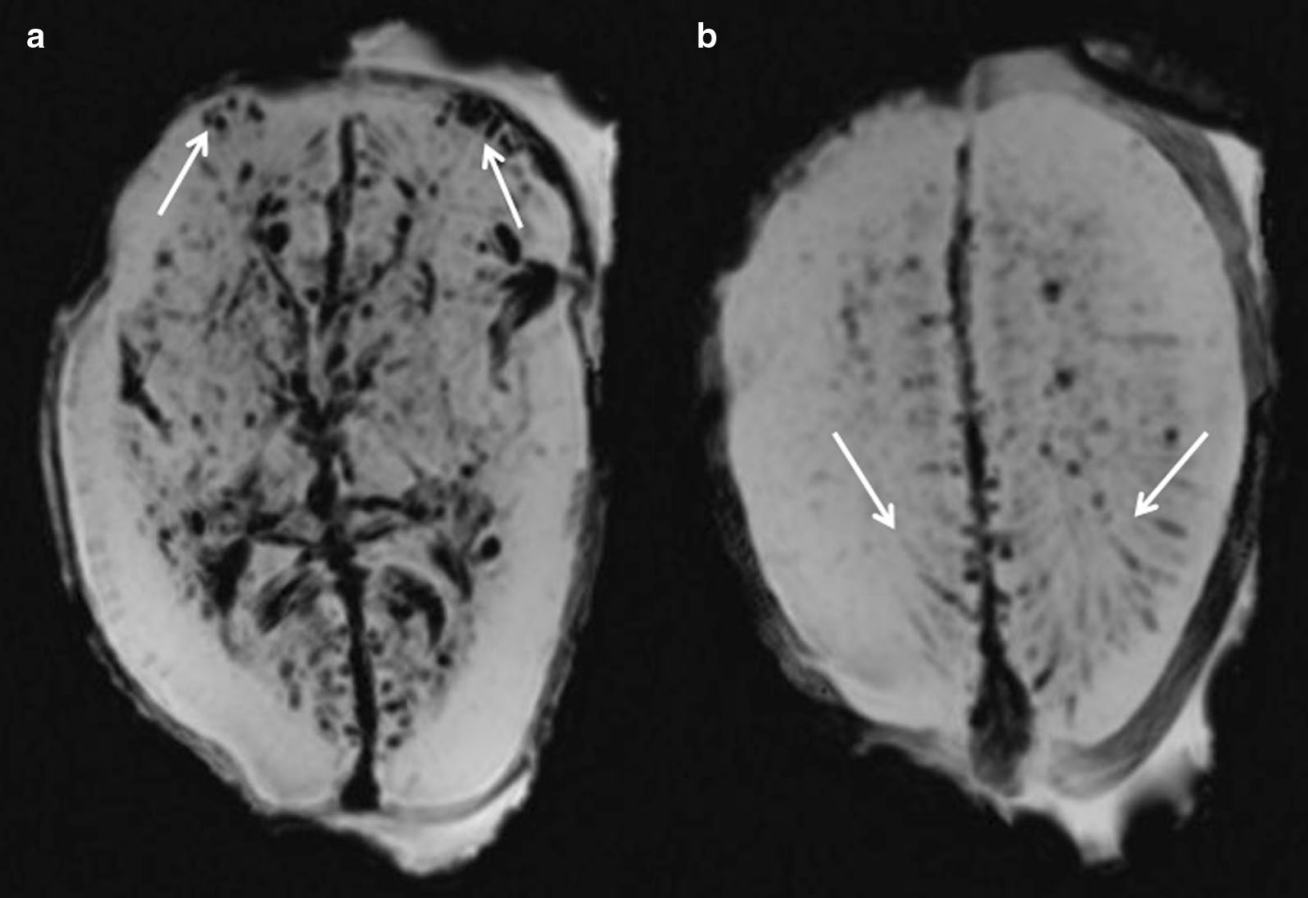
25-week foetus with normal cerebral lamination. Axial SWI images. **a** Appearance of the post-mortem brain with clots in cortical veins (arrows), **b** clots in intramedullary veins (arrows)

Several changes progressively take place after death and these should not be misinterpreted as pathological findings. Following death, there may be signal changes on T1W and T2W sequences due to autolysis, maceration, decreased temperature, and preservation techniques, among other causes, which can be erroneously interpreted as pathological. Loss of grey and white matter differentiation (Fig. 4), loss of normal high signal intensity in the posterior limb of the internal capsule, and white matter T2W prolongation, are imaging findings that can be seen in both normal post-mortem changes and in pre-mortem ischaemic injury [30]. As a result, it can be extremely difficult to differentiate between these two entities [8]. DWI sequences and the apparent diffusion coefficient (ADC) can also be altered by multiple causes, with maceration being the most common [21]. Venous stasis is a normal finding following death, but can also equally be misinterpreted as ante-mortem venous thrombosis [31] on SWI images (Fig. 5). Apparent tonsillar descent is a relatively frequent “normal” post-mortem finding, which may be attributed to cerebral oedema [31].

The whole birth process is traumatic for both mother and foetus, leading to MRI abnormalities that can be misinterpreted as pathological. These abnormalities include skull deformity, collapsed eyeballs, lens luxation, irregular indentations of cortical plate of frontal lobes, and small intraventricular haemorrhage without dilatation [32].

### Abnormal CNS findings

These are findings that are not encountered under normal conditions and often with a pathological background.

**Fig. 6:**
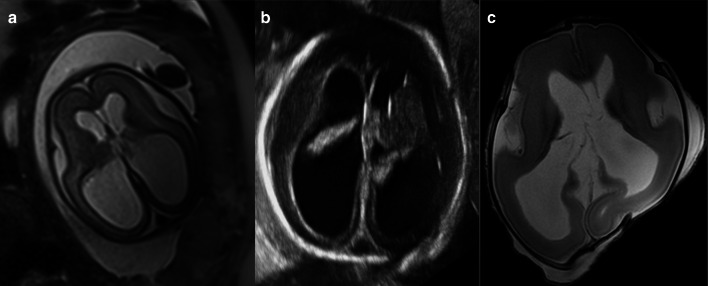
26-week, elective termination of pregnancy due to ventriculomegaly. **a** Intrauterine HASTE MRI sequence showing the ventricular dilation. **b** Intrauterine ultrasound shows the same findings and (**c**) axial post-mortem T2W MRI demonstrating the ventricular dilation

**Fig. 7:**
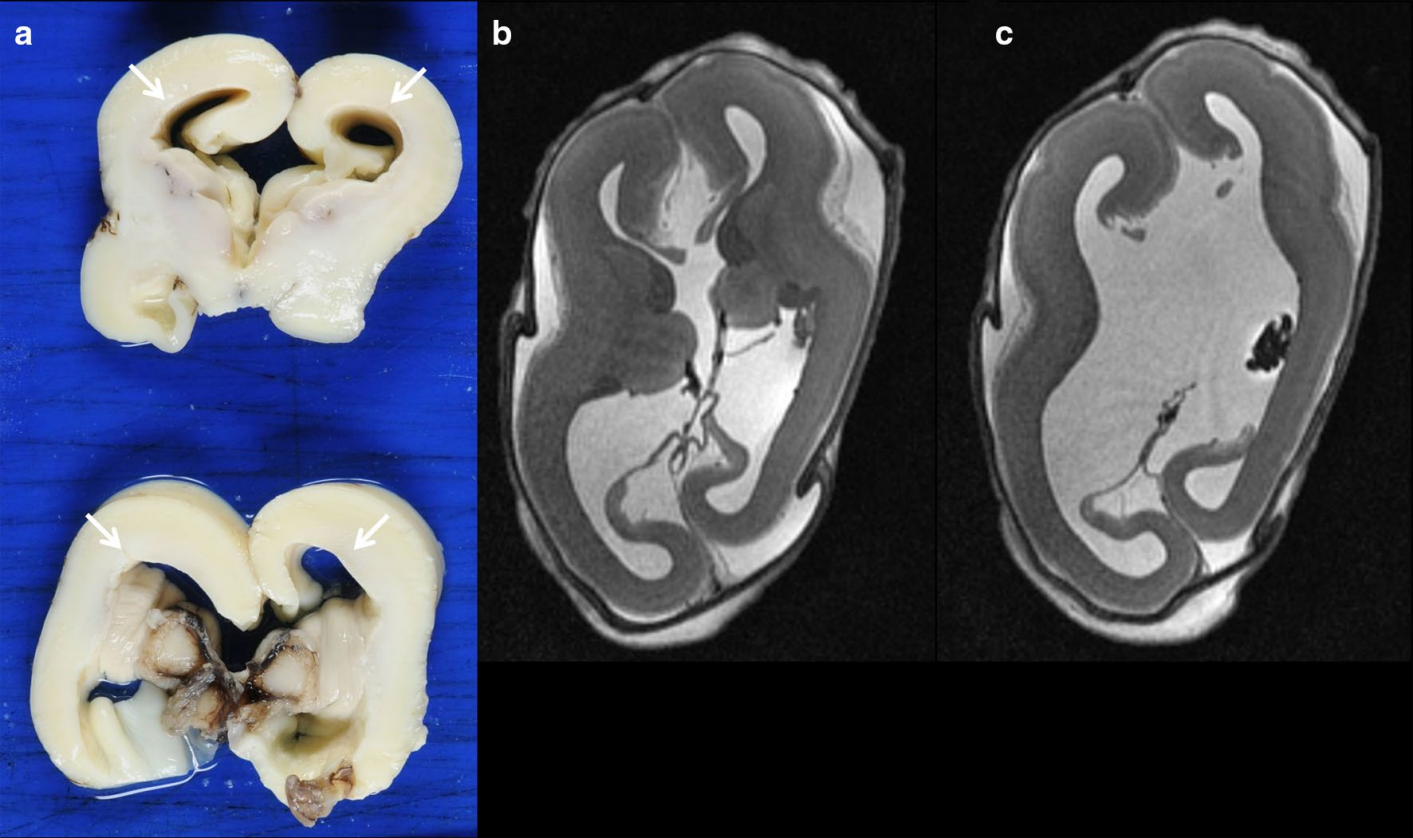
Ventriculomegaly in a 21-week foetus with elective termination of pregnancy due to CMV infection. **a** Pathology specimen showing ventriculomegaly (arrows) and absence of the corpus callosum, (**b**, **c**) axial T2W MRI demonstrating the same findings

**Fig. 8:**
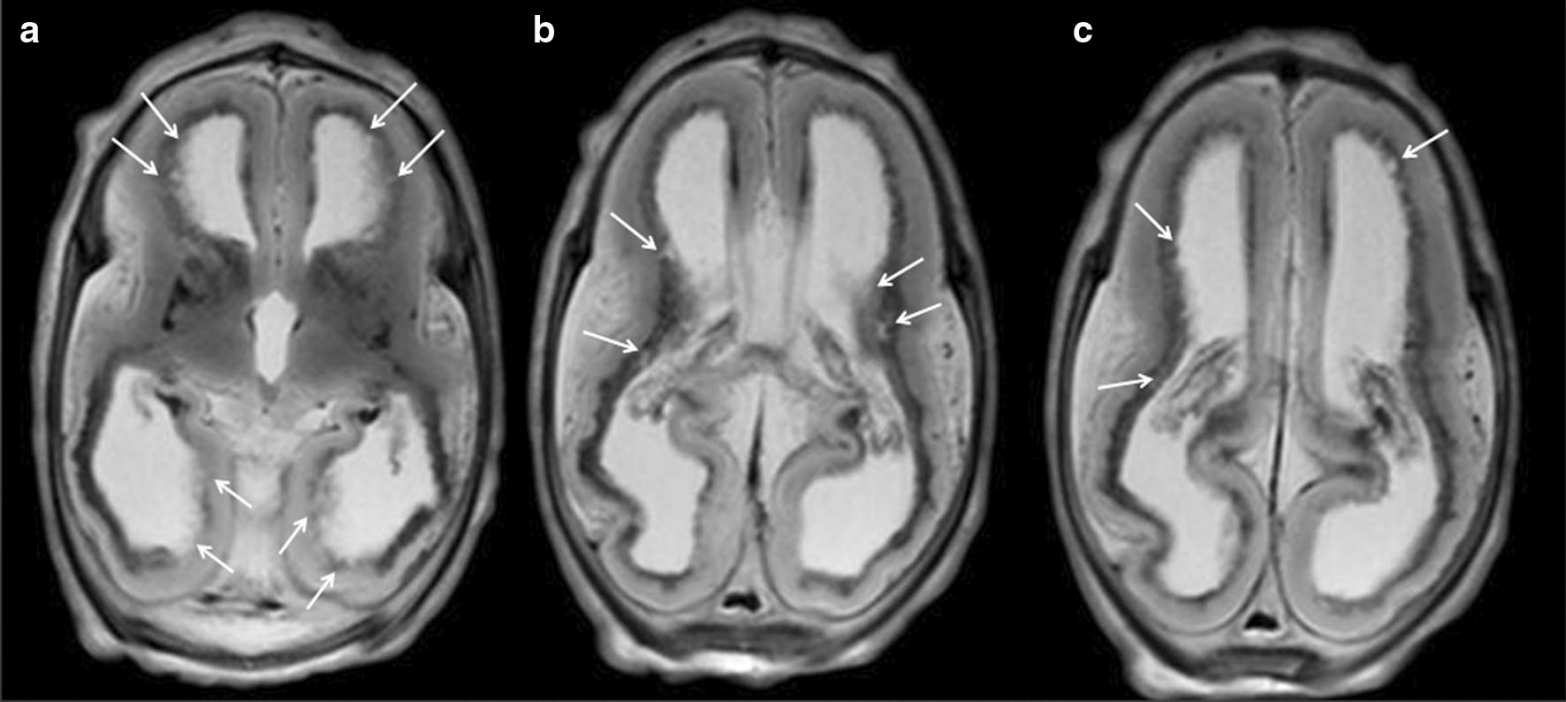
Cystic periventricular lesions in a 24-week foetus with elective termination of pregnancy due to CMV infection. **a**–**c** Axial T2W MRI showing ventricular dilatation and cystic periventricular lesions (arrows)

**Fig. 9:**
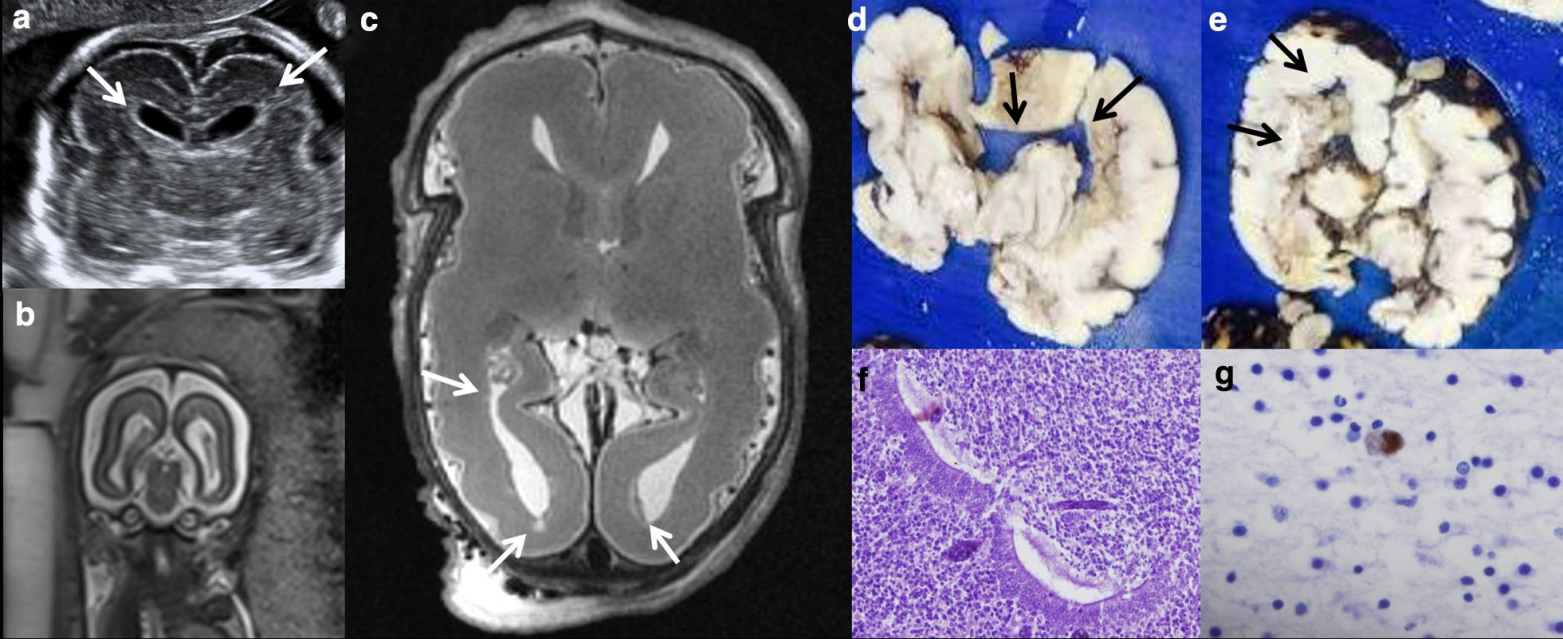
Cystic periventricular lesions in a 24-week foetus, elective termination of pregnancy due to CMV infection. **a** Intrauterine ultrasound demonstrating bilateral periventricular hyperecogenicity (arrows). **b** Intrauterine MRI showing bilateral ventriculomegaly. **c** Post-mortem MRI showing the ventricular dilation and periventricular cysts (arrows). **d**, **e** Pathology demonstrating the ventricular dilation (black arrows), macroscopic pathology does not demonstrate the subependymal cysts. **f** Ventriculitis and periventriculitis with disruption of the ependymal cells (H/E, ×60). **g** Positive cell against CMV immunohistochemistry (×60)

**Fig. 10:**
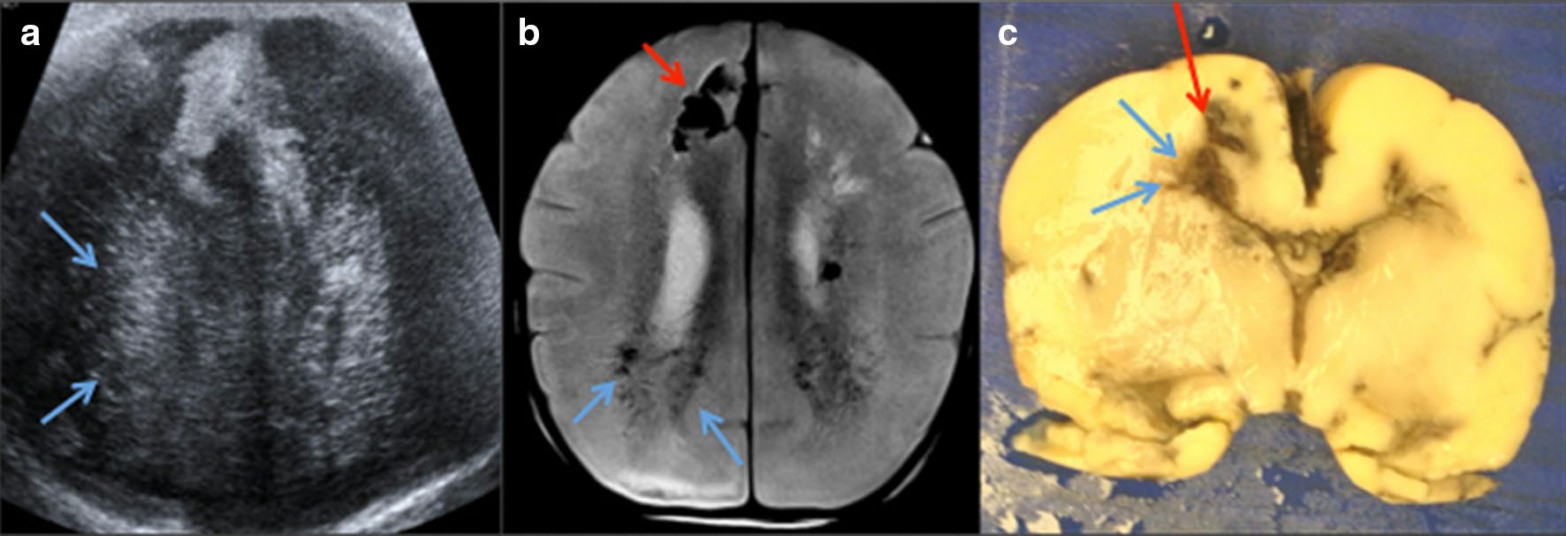
Intracranial haemorrhage in a 29-week premature newborn. **a** Ultrasound, (**b**) axial T2W MRI, (**c**) pathology showing periventricular (blue arrows) and frontal haemorrhage (red arrow)

**Fig. 11:**
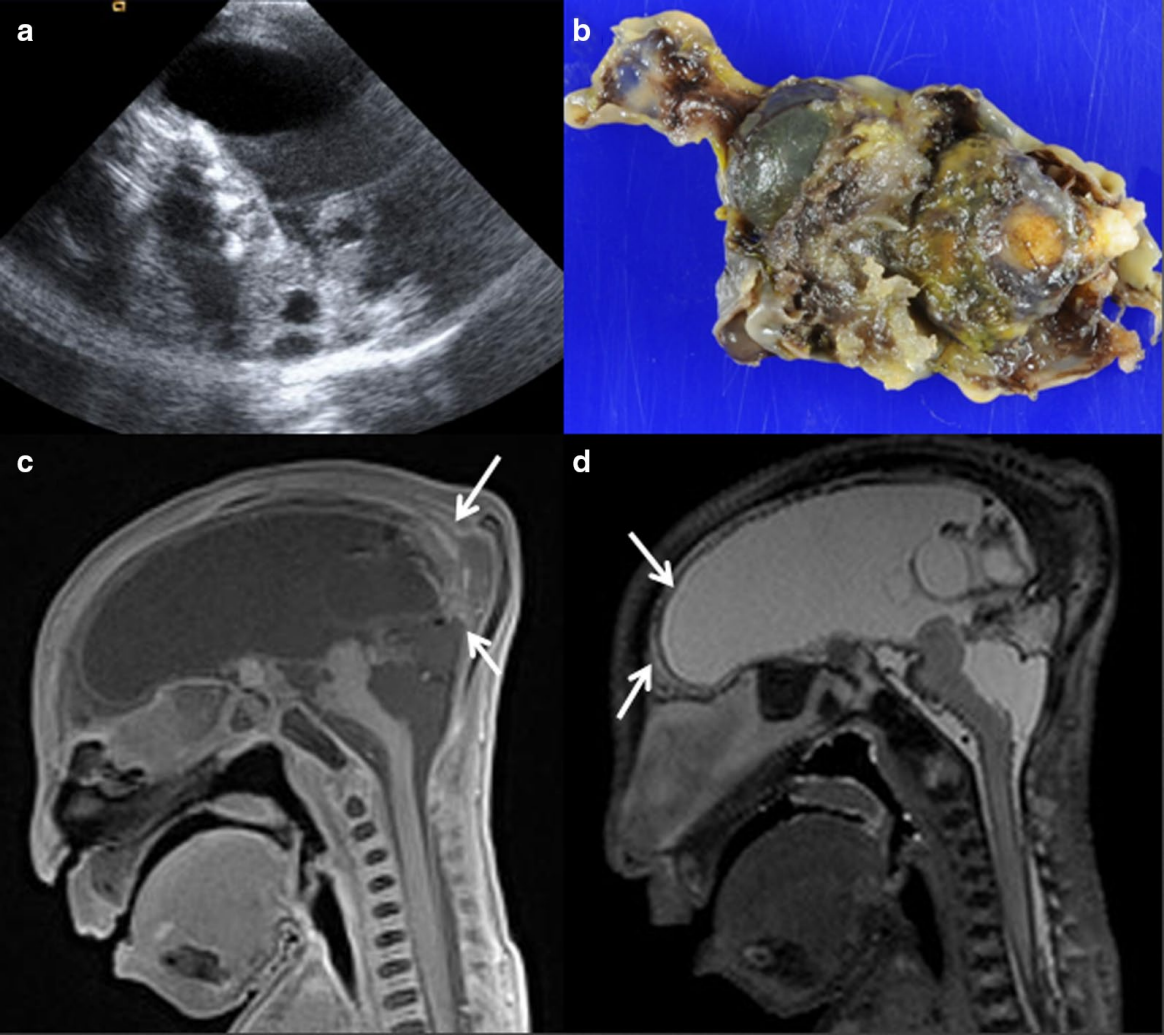
Hydranencephaly in a 30-week foetus. (**a**) Sagittal ultrasound. **b** Pathology specimen, microcephaly with cystic degeneration of the brain. **c** Sagittal T1W, (**d**) Sagittal T2W MRI showing brain parenchyma with residual cortical tissue (arrows)

**Fig. 12:**
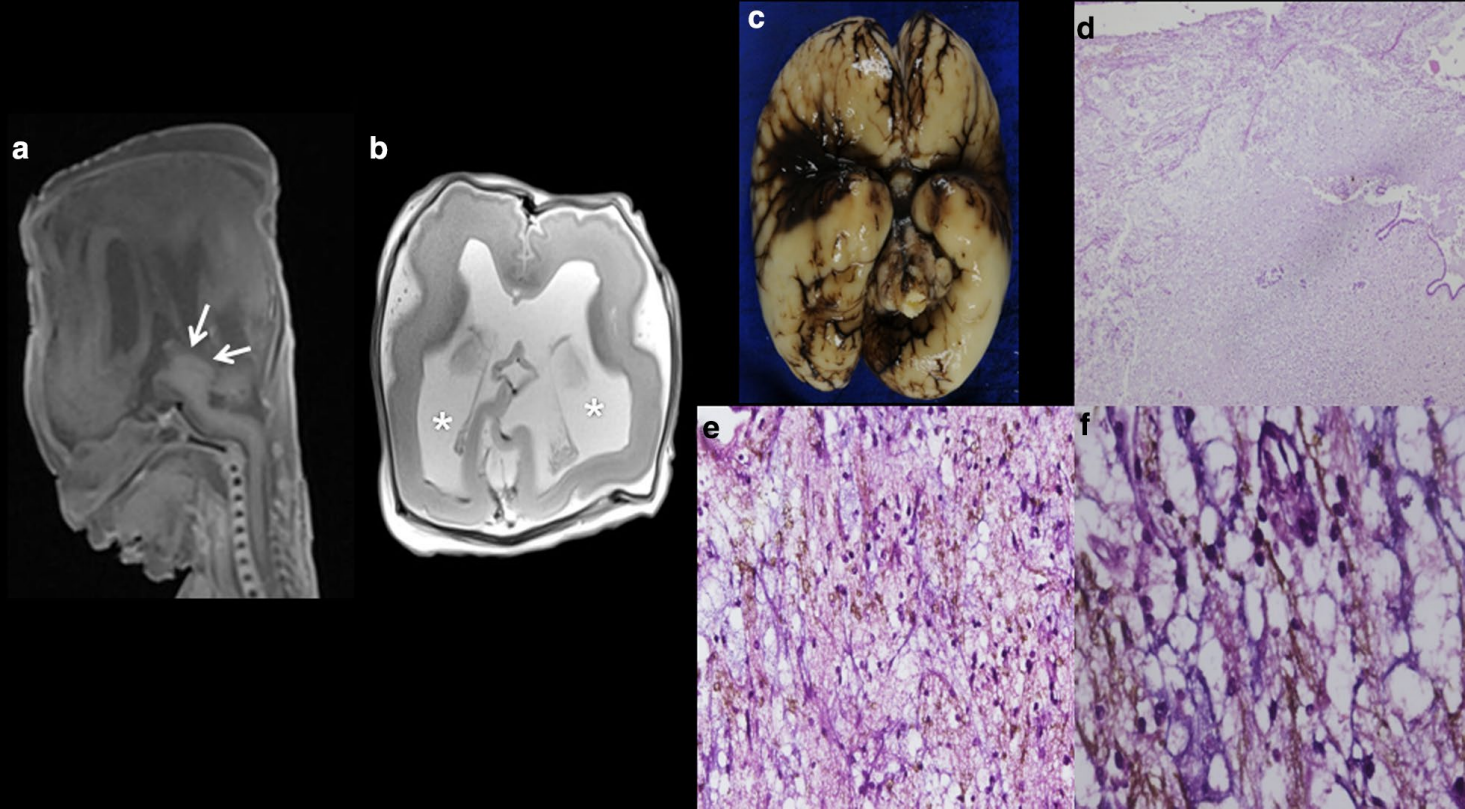
DNET in a 24-week foetus with elective termination of pregnancy. (**a**) Sagittal T1W MRI, (**b**) axial T2W MRI showing ventriculomegaly (asterisks) and a dysplastic mesencephalon (arrows), which were the cause of TOP. **c** Macroscopic specimen. **d** Tumour with DNET features involving the brainstem with infiltration of the Silvius aqueduct (H/E, ×4). **e**, **f** Double staining showing positivity for neurofibrils (immunohistochemistry against neurofibrils, in brown) intermingled with mucoid material (alcian blue, in blue) in the tumour (×40 and ×20)

**Fig. 13:**
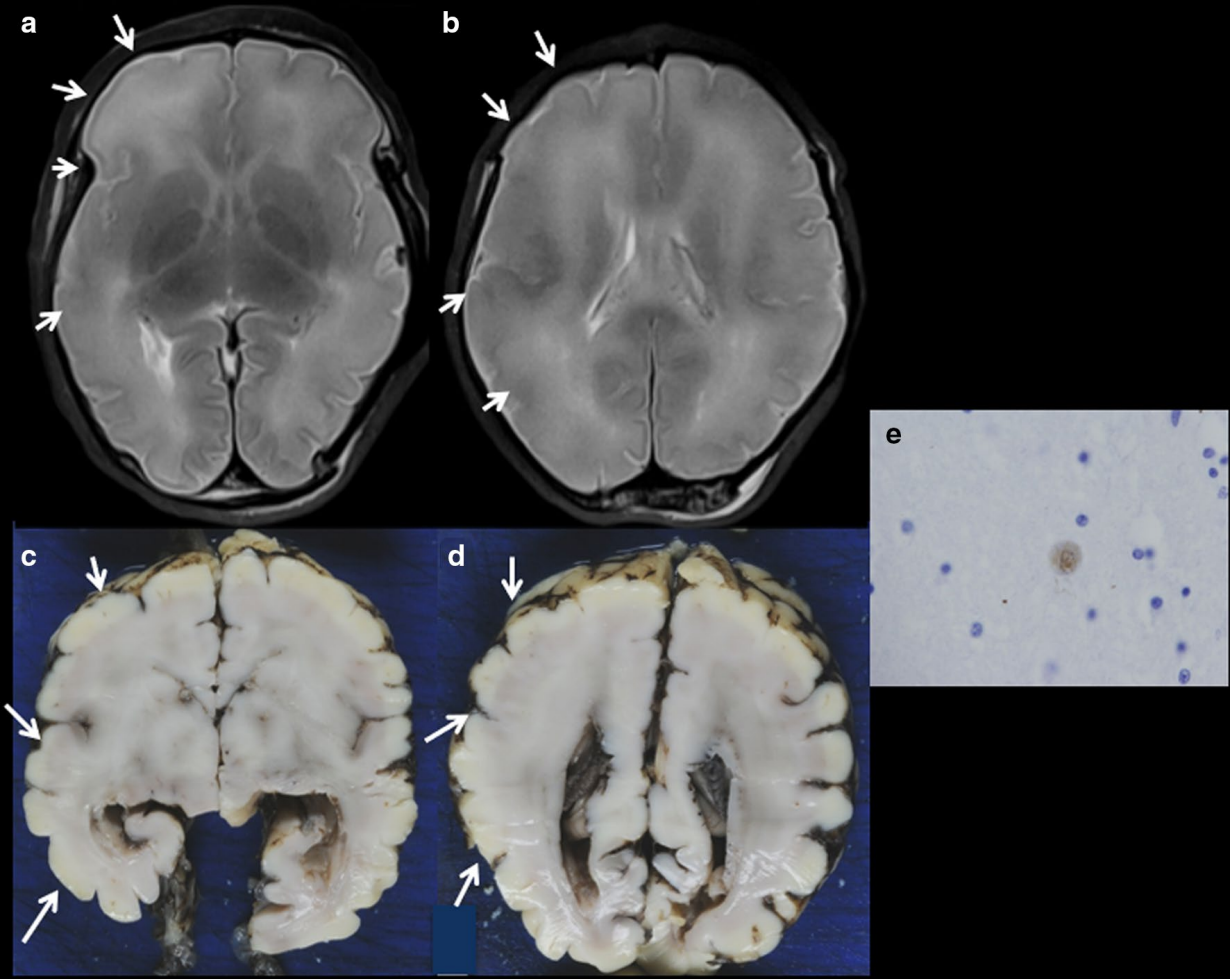
NMD in a 35-week foetus with elective termination of pregnancy due to CMV infection. **a**, **b** Axial T2W MRI showing a neuronal migration disorder in the right hemisphere (pachygyria), (arrows). **c**, **d** Macroscopic specimen showing pachygyria (arrows) and (**e**) positive cell against CMV immunohistochemistry (×60)

- *Ventriculomegaly* (Figs. 6, 7) This refers to enlargement of the cerebral ventricles, defined as a trans-trigone measurement of ≥ 10 mm at any stage of pregnancy [33, 34]. It is charactered as mild (10–12 mm), moderate (13–15 mm) or severe (> 15 mm) [35]. This condition is one of the most commonly detected foetal anomalies at the mid-trimester US study [36]. While foetal ventriculomegaly is a non-specific finding on foetal imaging, it can be associated with multiple aetiologies and a wide range of neurodevelopmental outcomes. In some cases it may be the only abnormal finding, leading to the term “isolated ventriculomegaly”, which has been related to an increased risk of aneuploidy [37] (particularly trisomy 21). Foetal ventriculomegaly can result from hydrocephalus, but it can also be secondary to poor brain development or loss of brain tissue [38]. Certain abnormalities related to foetal ventriculomegaly such as corpus callosum agenesis can be better detected on foetal MRI than US [39]. It is worth mentioning that ventriculomegaly can resolve in 50% of cases between the antenatal and post-mortem imaging period, possibly due to fluid shifts following death [12].
- *Periventricular white matter injury* This is defined as an increased T2-signal of the periventricular white matter. It is a non-specific finding that can be found in multiple pathological situations, but also as normal post-mortem physiological changes. Among the pathological causes, periventricular leukomalacia occurs as a result of hypoxic-ischaemic lesions resulting from impaired perfusion in the intervascular areas of the brain. The vascular supply to the brain changes with brain maduration. In the immature brain (before 36 weeks), ventriculopetal penetrating arteries extend inward from the surface of the brain to supply the periventricular regions; hence periventricular leukomalacia is the most common pathologic finding in hypoperfusion injury [40]. When evaluated by MRI, the acute phase of periventricular leukomalacia is characterised by areas of focal necrosis in the white matter, typically adjacent to the trigones of the lateral ventricles. Subsequently, these lesions acquire a cystic appearance. In the late stages, ventricular size increases due to loss of brain volume and narrowing of the corpus callosum [40].
- *Cystic periventricular lesions* (Figs. 8, 9) Many conditions can lead to foetal brain cysts, but the number and location of cysts can help to determine the underlying cause. The presence of small multiple subependymal periventricular and temporal pole cysts are highly suggestive of cytomegalovirus (CMV) infection. Among the “STORCH” infections (syphilis, toxoplasmosis, other infections, rubella, CMV and herpes simplex), CMV is the most common and has the highest mortality rate [31]. Periventricular calcification (difficult to assess by MRI), malformations of cortical development (lissencephaly and polymicrogyria) [41] and ventriculomegaly [42] can also be found in CMV infection. The subacute phase of periventricular leukomalacia of the foetus may also present with periventricular cystic lesions, corresponding to necrosis of the white matter, typically located adjacent to the trigones of the lateral ventricles [40]. Subependymal cystic lesions can also be found as sequelae of intraventricular haemorrhage [43]. Porencephalic cysts are other lesions originating from an insult to the foetal brain (hypoxic-ischaemic injury, intraparenchymal haemorrhage, infection, etc.). These are usually located in the cerebral hemispheres and typically communicate with the ventricular system [44].
- *Intracranial haemorrhage* (Fig. 10) The majority of intracranial haemorrhages are easily depicted on MRI, but differentiation between ante-mortem and post-mortem haemorrhage can be difficult. As mentioned above, small intraventricular bleeds in early gestation foetuses are likely to be considered a normal post-mortem physiologic MRI finding [45]. On the other hand, large haemorrhages are rare in older foetuses [31], and therefore tend to be considered as a potential cause of death. These are usually secondary to brain haemorrhagic infarctions. In the context of hypoxic-ischaemic encephalopathy, cerebral haemorrhage may be associated with periventricular white matter injury [40]. Haemorrhages may also be found in association with other inflammatory processes or infections such as CMV [42]. When intraventricular haemorrhage is present, clots are visualised within the ventricles and may be accompanied by ventriculomegaly [43, 46]. As mentioned above, intracranial haemorrhages can result in cystic sequelae (subependymal cysts and/or porencephalic cysts) [43].
- *Hydranencephaly* (Fig. 11) This is a rare encephalopathy characterised by the absence/destruction of the cerebral hemispheres, which are replaced by a cavity filled with cerebrospinal fluid and residual brain tissue. It is usually sporadic and its cause remains unknown. On MRI it manifests as extensive areas of cystic encephalomalacia, relatively symmetrical and without mass effect [47].
- *Congenital brain tumours* Foetal intracranial tumours are rare, and they are most often located in the supratentorial region. Teratomas are the most frequent foetal CNS tumours, accounting for approximately half of all reported cases [48, 49]. They are followed in frequency by neuroepithelial tumours and lipomas. Dysembrioplastic neuroepithelial tumour (DNET) (Fig. 12) is a benign tumour (WHO Grade I) of neuroepithelial origin that arises from cortical or deep grey matter, and it is mostly located in the temporal lobe (62%) [50]. Although the vast majority of DNET are confined to the cortical grey matter, they can also arise from the caudate nucleus [51], cerebellum or pons [52]. They are characterised by a mixture of astrocytes and oligodendroglial elements, in association with “floating neurons” and mucinous degeneration [50].
- *Neuronal migration disorders (NMD)* (Fig. 13) These are also defined as “malformations of cortical development” and comprise a heterogeneous group of situations in which there is an abnormal cortical organisation. These alterations may occur sporadically, be associated with genetic alterations or be linked to an insult during intrauterine neuronal development such as CMV infection [31]. Lissencephaly consists of cortical thickening associated with a variable lack of normal brain circumvolutions, and although it can be diagnosed from the onset of sulcation, it is more obvious in the third trimester [53]. Heterotopia of grey matter refers to ectopic neuronal clusters most often located subependymal in the lateral ventricles [54], although they can be located anywhere in the cerebrum or cerebellum. Polymicrogyria consists of small supernumerary gyri, which if not extensive can be very difficult to detect [47]. Schizencephaly presents as a cleft demarcated by polymicrogyric grey matter that extends from the cortical surface to the ventricular margin [47].

### Disadvantages of post-mortem MRI

One disadvantage of post-mortem foetal MRI is that on many occasions the alterations detected do not explain the cause of death (for example in cases of ventriculomegaly, agenesia of corpus callosum, etc.), as some macroscopic findings are non-specific. Moreover, transporting foetuses in the best conditions to reference centres can also be a challenge. Assessment of foetal CNS abnormalities is difficult, and interpretation of foetal studies requires familiarization with normal and abnormal findings in post-mortem MRI studies as well as the strengths and limitations of the imaging studies.

## Future prospects

Post-mortem perinatal imaging has many opportunities for further research. Indeed, post-mortem MRI and autopsy have a high macroscopic correlation and may be complementary techniques for the evaluation of the CNS [8, 9, 31, 45, 55]. Therefore, as suggested by other authors, the combination of these techniques could change the approach to the autopsy process itself [8]. Post-mortem MRI may be routinely performed before autopsy in cases of foetal death. In cases of technically complicated autopsies due to gestational age or possible autolytic changes, previous post-mortem MRI may optimise diagnostic information. In other cases, depending on the post-mortem MRI findings, CNS conventional autopsy may not be necessary, or a minimally invasive autopsy could be performed.

In our centre we reported a case in which the autopsy was not assessable due to advanced brain maceration, but the previous post-mortem foetal MRI was able to detect periventricular cystic lesions suggesting CMV infection. This situation has been previously reported in the literature, demonstrating that post-mortem MRI can provide significant information in up to 50% of foetuses in which conventional autopsy is not assessable due to maceration and/or autolysis [56]. This could be an interesting topic to consider in future approaches on the utility of post-mortem foetal MRI.

Future studies should evaluate the cost-effectiveness of routinely employing post-mortem MRI combined with autopsy in the study of the CNS in cases of foetal death. The implementation of structured reports may help to standardise nomenclature and optimise the quality of radiological reports.

## Conclusion

Post-mortem MRI provides useful morphologic information of the CNS in dead foetuses, showing a close correlation with autopsy findings, and could be a valid alternative when autopsy cannot be performed. In the near future, post-mortem foetal MRI could be routinely added alongside autopsy in the study of CNS in perinatal deaths, as both techniques can be complementary. It is mandatory for radiologists to understand the imaging characteristics of post-mortem foetal MRI in order to achieve the most optimal results.

## Data Availability

The material used are part of this manuscript.

## Abbreviations

ADC: Apparent diffusion coefficient
CISS: Constructive interference steady state
CMV: Cytomegalovirus
CNS: Central nervous system
DNET: Dysembrioplastic neuroepithelial tumour
DWI: Diffusion weighted imaging
IUS: Intrauterine sonography
MRI: Magnetic resonance imaging
NMD: Neuronal migration disorder
SWI: Susceptibility weighted imaging
T: Tesla
T1W: T1-weighted
T2W: T2-weighted
TOP: Termination of pregnancy
US: Ultrasound
